# Targeting replication stress to tackle cancer stem cells

**DOI:** 10.1038/s41419-021-03609-8

**Published:** 2021-03-24

**Authors:** Lorenzo Galluzzi

**Affiliations:** 1 Department of Radiation Oncology, Weill Cornell Medical College, New York, NY USA; 2 Sandra and Edward Meyer Cancer Center, New York, NY USA; 3 Caryl and Israel Englander Institute for Precision Medicine, New York, NY USA; 4 Department of Dermatology, Yale School of Medicine, New Haven, CT USA; 5 Université de Paris, Paris, France

**Keywords:** Cancer stem cells, Colorectal cancer, Cell death

Most (if not all) solid neoplasms are characterized by at least some degree of intratumoral heterogeneity, largely reflecting the Darwinian co-evolution of multiple cell populations in the context of various selective pressures that differ across tumor regions and fluctuate over time^1^. Specifically, the malignant cell compartment of colorectal carcinoma (CRC) comprises a population of cells that self-renew as they generate a relatively differentiated progeny in support of disease progression^2^. As compared with their more-differentiated counterparts, these cancer stem cells (CSCs) are resistant to a variety of commonly employed anticancer agents, thus (1) standing out as major responsible for treatment failure and poor disease outcome, and (2) representing attractive targets for the development of novel therapeutic approaches^3^. As the superior resistance of CSCs to treatment (at least partially) stems from a hyper proficient DNA-damage response (DDR), considerable interest has been generated by the possibility of harnessing DDR-targeting agents to eradicate CSCs^4^. However, the intrinsic heterogeneity of the CSC compartment and its ability to rapidly activate additional resistance mechanisms has limited the success of such an approach so far. Moreover, the ability of CSCs to engage in the DNA replication stress (RS) response, the oncosuppressive mechanism elicited in the S phase of the cell cycle by problems of the DNA replication fork (as those induced by some chemotherapeutics)^5^, has been the subject of debate. Recent findings from Manic et al.^6^ not only demonstrate that CSCs from multiple patients with CRC can proficiently activate the RS response, but also identify combinatorial approaches targeting the DDR that can be harnessed to eradicate treatment-resistant CSCs based on synthetic lethality.

As most CSCs from patients with CRC require the key RS response mediator checkpoint kinase 1 (CHEK1, best known as CHK1) for survival^7^, Manic and collaborators harnessed prolonged, repeated exposure to a pharmacological CHK1 inhibitor (CHK1i) to relieve CSCs from RS response addition, ultimately obtaining CHK1i-resistant CSCs. As compared with their CHK1i-sensitive counterparts, CHK1i-resistant CSCs also exhibited increased resistance to inhibition of the CHK1-activating kinase ATR serine/threonine kinase (ATR) and to clinically employed chemotherapeutics that trigger the RS response, such as irinotecan, 5-fluororucil, and oxaliplatin. Conversely, CHK1i-resistant CSCs resembled their CHK1i-sensitive counterparts in responsiveness to inhibitors of other DDR kinase-like ATM serine/threonine kinase (ATM) and CHK2. Moreover, both CHK1i-resistant and CHK1i-sensitive CSCs exhibited a functional RS response, as demonstrated by their accumulation at metaphase (rather than in the S phase) upon exposure to the RS inducer hydroxyurea (HU) followed by spindle poison nocodazole (N). However, CHK1i-resistant CSCs displayed limited activation of the RS response at baseline and upon exposure to a CHK1i, correlating with increased levels of the DDR component poly(ADP-ribose) polymerase 1 (PARP1)^6^.

A pharmacological PARP1 inhibitor (PARP1i) neither affected the ability of CHK1i-resistant CSCs to mount an efficient RS response upon exposure to HU + N, nor it impaired baseline RS functions, as assessed by the flow cytometry-assisted quantification of fork breakdown, or cell survival. Conversely, concomitant exposure to a PARPi and a CHK1i provoked severe RS in CHK1i-resistant (but not CHK1 sensitive) CSCs, correlating with highly decelerated fork progression and accumulation of single-stranded (ss)DNA (a marker of DNA degradation at active forks). Moreover, PARP inhibition synergized with CHK1 inhibition at causing the demise of CHK1i-resistant CSCs, especially when achieved with agents that strongly trap PARP1 on damaged DNA, such as olaparib and talazoparib. Consistent with this notion, olaparib plus a CHK1i robustly controlled the growth of CHK1i-resistant CSCs xenografted in immunodeficient mice, whereas neither agent had in vivo anticancer effects when employed as monotherapy. Moreover, CSCs from patients with CRC failed to acquire resistance to CHK inhibition when exposed to a CHK1i in a prolonged and repeated manner in the presence of a PARPi, resulting instead in the near to complete eradication of CSCs as a result of accrued RS^6^.

Finally, Manic and colleagues investigated the sensitivity of CHK1i-resistant CSCs exhibiting PARP1 upregulation to other DDR-targeting agents, revealing a synergistic effect from combined MRE11 homolog, double-strand break repair nuclease (MRE11), and RAD51 recombinase (RAD51) inhibition, but virtually no effects from MRE11 and RAD51 inhibitors employed as standalone therapeutics. Such a synergy correlated with the ability of MRE11 and RAD51 co-inhibition to compromise the RS response of CHK1i-resistant CSCs exposed to HU + N, culminating with cell cycle progression up to abortive mitosis and caspase 3-driven cell death^6^.

**Fig. 1:**
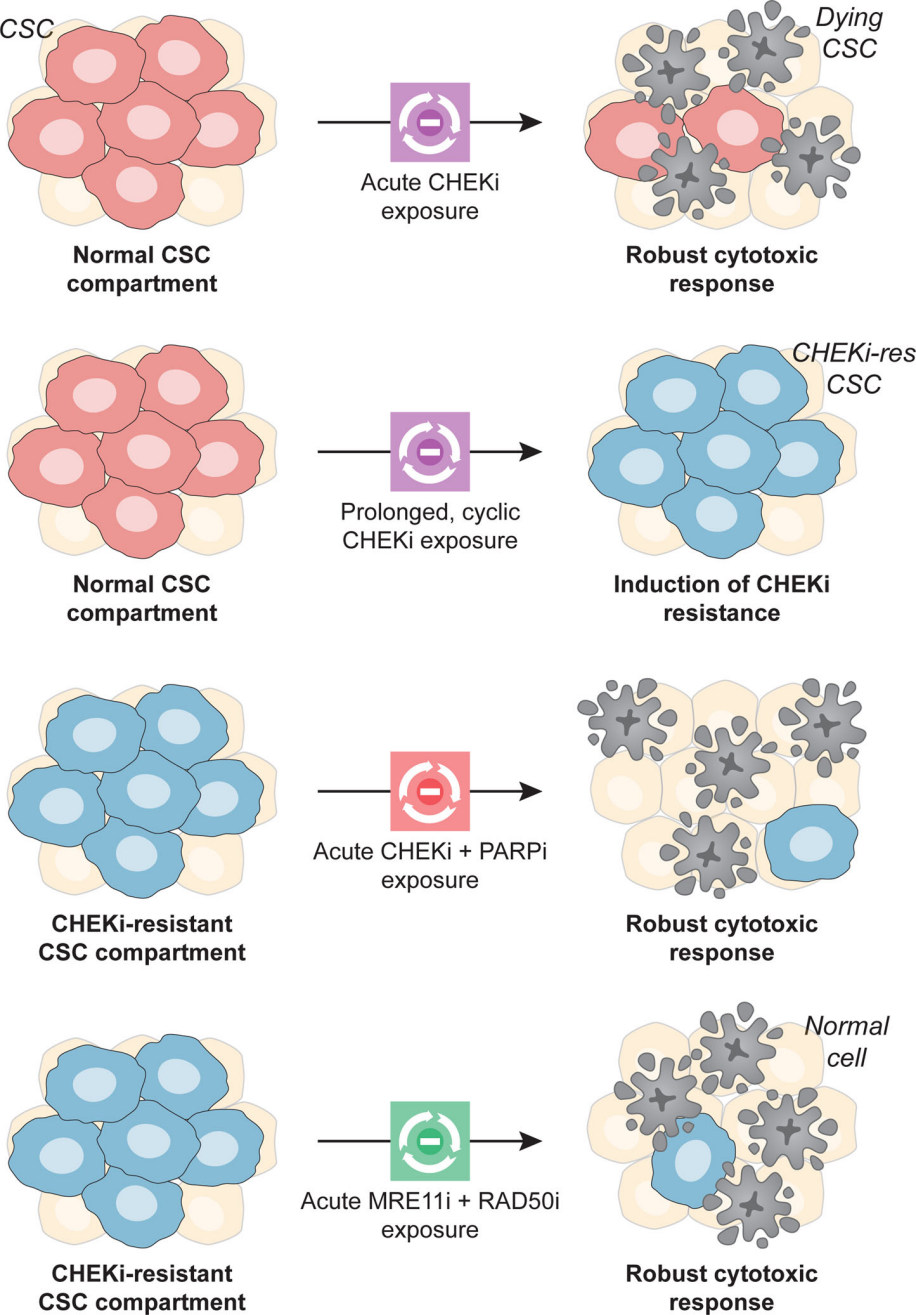
Targeting the DNA-damage response to tackle the CSC compartment. Cancer stem cells (CSCs) are generally resistant to DNA-damaging agents, at least in part owing to a hyper proficient DNA-damage response, but often rely on checkpoint kinase 1 (CHEK1, best known as CHK1), a major signal transducer in the DNA replication stress response, for survival. Conversely, CSCs with innate (not shown) or acquired resistance to CHK1 inhibitors (CHK1is) exhibit poly(ADP-ribose) polymerase 1 (PARP1) upregulation and require the combined functions of CHK1 plus PARP1, and MRE11 homolog, double-strand break repair nuclease (MRE11) plus RAD51 recombinase (RAD51) for survival. Thus, CHK1 plus PARP1 and MRE11 plus RAD51 identify two main hubs for combinatorial therapeutic approaches against CSCs based on the principle of synthetic lethality. *MRE11i* MRE11 inhibitor, *PARP1i* PARP1 inhibitor, *RAD51i* RAD51 inhibitor.

Taken together, these findings delineate several alterations in the DDR of CSCs that may be harnessed from a therapeutic perspective (Fig. 1), as agents targeting CHK1, PARP1, and RAD51 are either approved for use in cancer patients or under clinical development^8^. As most DDR-targeting drugs have been associated with the spillage of DNA molecules in the cytosol and consequent secretion of type I interferon (IFN) downstream of cyclic GMP-AMP synthase activation^9^, it will be interesting to investigate whether CHK1i-resistant CSCs emit immunogenic signals as they succumb to combined CHK1 and PARP1 (or MRE11 and RAD51) inhibition, as well as whether radiation therapy (a potent activator of the DDR and type I IFN secretion)^10^, can be harnessed in combination with any of these strategies to eradicate the CSC compartment. Irrespective of these hitherto untested possibilities, the therapeutic vulnerabilities identified by Manic and colleagues stand out as promising targets to develop CSC-directedtherapeutic regimens with clinical applications.
